# Montana

**Published:** 1897-04

**Authors:** 


### CONTROL WORK.

*Montana.* State Veterinarian Knowles denies the existence of scab in sheep in his State. The only outbreak was through some Wyoming sheep brought into the State. Governor Smith, by proclamation, will quarantine against the following States owing to the belief that scab prevails among their sheep: Oregon, Nevada, California, Washington, Wyoming, Idaho, Colorado, Utah, Oklahoma, and New Mexico. Each county of Montana has a sheep inspector.

